# Synthesis and ^64^Cu‐Radiolabeling Strategies of Small Organic Radioconjugates Based on the AMD070 Scaffold

**DOI:** 10.1002/cmdc.202500243

**Published:** 2025-06-14

**Authors:** Marie M. Le Roy, Patricia Le Saëc, Michel Chérel, Alain Faivre‐Chauvet, Thibault Troadec, Raphaël Tripier

**Affiliations:** ^1^ Univ. Brest, UMR CNRS 6521 CEMCA 6 avenue Le Gorgeu 29200 Brest France; ^2^ Univ. Angers, Nantes Univ., Inserm, CNRS, CRCI2NA, CHU Nantes F‐44007 Nantes France; ^3^ Institut de Cancérologie de l’Ouest F‐44800 Saint‐Herblain France; ^4^ Nuclear Medicine Department University Hospital 44000 Nantes France

**Keywords:** chelators, coordination, copper‐64, CXCR4, positron emission tomography imaging

## Abstract

CXCR4 is a transmembrane receptor overexpressed in a large variety of cancer cells. In addition to classical antibody‐ and peptide‐targeting for the development of positron emission tomography (PET) radiopharmaceuticals, this receptor possesses a range of small organic inhibitors that can be exploited. These are based mainly on nitrogen‐rich scaffolds, such as AMD070, and cyclic polyamines, such as cyclam, in the AMD3100 skeleton. The latter has been explored as a direct ^64^Cu chelator, but examples of CXCR4 PET imaging with small organic targeting units are still scarce in the literature. Herein, the synthesis of two novel CXCR4‐directed radiopharmaceuticals is described, combining the AMD070 scaffold as a targeting unit and bifunctional te1pa macrocycle as a strong ^64^Cu chelator. The synthesis of the conjugates and optimization of ^64^Cu‐radiolabeling are presented, opening the way for future *in vitro* and *in vivo* studies.

## Graphical abstract

Two novel ^64^Cu‐radioconjugates have been prepared, based on the AMD070 scaffold as a targeting unit and cyclam‐based te1pa chelator, for future applications in positron emission tomography imaging. Synthesis of ligands and their ^64^Cu‐radiolabeling are described herein.

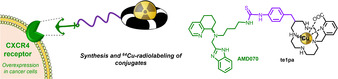

## 1 Introduction

**Scheme 1:**
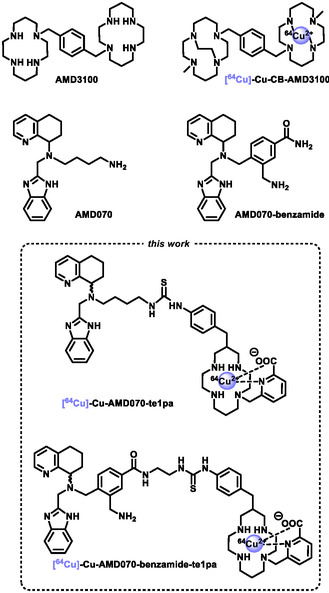
Small synthetic CXCR4 inhibitors discussed in this work.

CXCR4 is a transmembrane chemokine receptor, involved in lymphocyte chemotaxis and hematopoiesis in healthy tissues,^[^ ^1^ ^]^ but is also associated to autoimmune and inflammatory diseases and to the infection by human immunodeficiency virus.^[^ ^2^, ^3^ ^]^ It is overexpressed in a large variety of cancers^[^ ^4^, ^5^ ^]^ and hematological malignancies, such as B‐cell lymphoma and multiple myeloma.^[^ ^6^ ^]^ Therefore it has emerged as a target of choice in preclinical and clinical research for the treatment of these pathologies with antibodies^[^ ^7^ ^]^ and peptides.^[^ ^8^, ^9^, ^10^ ^]^ In addition, those biomolecules have also been extensively used in nuclear medicine as targeting units in radiopharmaceutical compounds.^[^ ^11^ ^]^ In particular, ^68^Ga‐Pentixafor is currently in clinical trials as a CXCR4‐targeting positron emission tomography (PET) radiotracer,^[^ ^12^ ^]^ alongside its ^177^Lu‐Pentixather counterpart for *β*‐radiotherapy.^[^ ^13^, ^14^ ^]^ However, these biomacromolecules can have several drawbacks, with usually long biodistribution times that are not suitable to all applications, in addition to demanding synthesis and storage. Moreover, the only FDA‐approved therapeutic CXCR4‐inhibitors to date are two small synthetic organic compounds, namely **AMD3100** ^[^ ^15^, ^16^, ^17^ ^]^ and **AMD070** (**Scheme** **1**).^[^ ^18^, ^19^, ^20^ ^]^ **AMD3100** is a *N*,*N*′,biscyclam with a *para*‐xylene spacer between the two cyclam units, whereas **AMD070** has a nitrogen‐rich scaffold composed of tetrahydroquinoline and benzimidazole entities on a butylene diamine chain. Their interaction with the CXCR4 receptor has been clearly evidenced, showing that multiple protonations of their amine functions at physiological pH, forming ammonium groups, trigger strong H‐bonding interactions with aspartate and glutamate fragments from different transmembrane domains of the receptor.^[^ ^21^, ^22^, ^23^, ^24^ ^]^ This feature provides excellent half‐maximal inhibitory concentrations (IC_50_) in the low nanomolar range (<15 nM),^[^ ^25^, ^26^ ^]^ similar to the one of cyclic peptides used as targeting units in pentixafor for instance (IC_50_ = 6 nM for the CPCR4.2 peptide alone). **AMD3100** structure is based on the cyclam polyazamacrocycle, long‐known for the stable coordination of small metallic cations^[^ ^27^, ^28^ ^]^ and copper(II) in particular.^[^ ^29^, ^30^ ^]^ Therefore, the CXCR4‐affinity of **AMD3100** complexes with various metallic cations has been investigated, and this strategy demonstrated a significant improvement of the inhibition properties versus “empty” **AMD3100**. The compound was then radiolabeled with ^64^Cu for an application as a CXCR4‐targeted PET imaging probe,^[^ ^31^ ^]^ keeping in mind that the ^64^Cu/^67^Cu theranostic pair of emitters (*β*+ for PET and *β*− for internal radiotherapy, respectively) could allow future translation to therapeutic applications as well. However, release of free ^64^Cu *in vivo* was observed with accumulation in the liver, owing to the poor kinetic inertness of the cyclam–Cu(II) complex. Inspired by the rich Cu(II) coordination chemistry of cyclam analogs, and rigid cross‐bridged structures in particular,^[^ ^32^, ^33^ ^]^ Archibald et al. designed rigid biscyclams that could ultimately be radiolabeled with ^64^Cu ([^**64**^ **Cu**]**Cu‐CB‐AMD3100**, Scheme 1),^[^ ^34^, ^35^ ^]^ showing a strong potential as a targeted PET probe, with excellent stability and CXCR4‐recognition properties *in vivo*. Regarding the **AMD070** counterpart, that does not have the chelating abilities to strongly bind copper(II) cations, no radiolabeled version for PET imaging has been described to date. Herein, based on our previous research on robust ^64^Cu chelators,^[^ ^36^, ^37^, ^38^, ^39^ ^]^ we sought to graft a dedicated macrocycle on the **AMD070** skeleton to allow its ^64^Cu‐labeling for future *in vitro* and *in vivo* studies. Such assemblies should present fast biodistribution that could be particularly suited for the future diagnosis of the hematological malignancies, such as B‐cell lymphoma and multiple myeloma, as a first approach.

The te1pa chelator, as demonstrated previously, meets all criteria for *in vivo* applications, from fast radiolabeling to outstanding inertness (in competitive media and serum) and good clearance *in vivo*.^[^ ^36^, ^37^ ^]^ Its bifunctional derivative bearing an isothiocyanate function has also demonstrated its suitability for grafting on targeting molecules and *in vivo* inertness in mice.^[^ ^40^, ^41^ ^]^ This derivative can be directly grafted on the distal primary amine of **AMD070** leading to the **AMD070‐te1pa** conjugate through the formation of a thiourea link (Scheme 1). However, as this amino group is involved in the interactions with CXCR4,^[^ ^24^ ^]^ targeting properties of the AMD070 fragment could be altered. Therefore, we also envisaged here an alternative version, based on the **AMD070‐benzamide** analog, that has an excellent IC_50_ (10 nM range),^[^ ^26^ ^]^ but preserves a primary amine function with the same chain length in the corresponding conjugate **AMD070‐benzamide‐te1pa**. In the current study, we present the synthesis of the two novel conjugates and the development of their ^64^Cu radiolabeling to consider future CXCR4‐targeting experiments.

## 2 Results and Discussion

### 2.1 Synthesis of the Conjugates

**Scheme 2:**
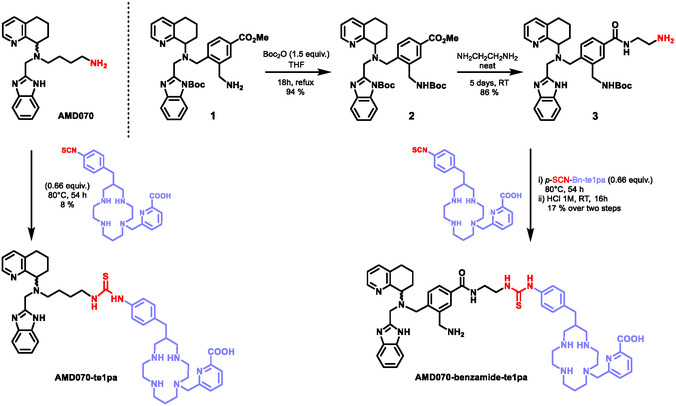
Synthesis of **AMD070‐te1pa** and **AMD070‐benzamide‐te1pa** conjugates.

**AMD070** was prepared according to literature.^[^ ^42^ ^]^ For the functionalization of the **AMD070‐benzamide** compound, an additional ethylamine spacer providing a primary amine necessary for coupling had to be introduced, with concomitant Boc‐protection of the benzylamine fragment (**Scheme** **2**). Thus, compound **1** was prepared via a procedure adapted from literature (see Supporting Information),^[^ ^43^ ^]^ and reacted with di‐*tert*‐butyl dicarbonate (Boc_2_O), providing **2** with nearly quantitative yield. Subsequent reaction with ethylenediamine (EDA) for 5 days provided **3** in 86% yield. Noteworthy, this reaction was conducted in diluted conditions (0.1 mmol mL^−1^) with very large excess EDA (neat) and at room temperature to prevent formation of dimeric species from reaction on both amino groups of EDA. Both new compounds were fully characterized by ^1^H and ^13^C nuclear magnetic resonance (NMR) spectroscopy as well as high‐resolution mass spectrometry (HRMS, Electrospray ionization). In particular, diagnostic signals of the ethylene spacer in **3** are observed in ^13^C NMR spectroscopy with chemical shifts of 43.4 and 42.3 ppm in CD_3_OD, as well as the one of the sp^2^ carbon atom from the amide function at 170.1 ppm (see Supporting Information). Surprisingly, the Boc protecting group on the benzimidazole fragment was cleaved during this last step, as evidenced in ^1^H NMR spectroscopy with the presence of a sole broad singlet accounting for nine protons from the *tert*‐butyl group on the distal amine, at 1.45 ppm.

The coupling conditions with ***p*** **‐SCN‐Bn‐te1pa** (20 mg, 0.024 mmol, trifluoroacetic acid (TFA) salt), prepared according to the literature procedure,^[^ ^44^ ^]^ were investigated first on **AMD070**, with a screening of solvent and base combinations, reaction time, and temperature (**Table** **1**).

**Table 1:** Conditions screening for the coupling reaction of AMD070 with *p*‐SCN‐Bn‐te1pa.

AMD070 (Equiv.)	Solvent	Base (exc.)	*T* [°C]	*t* [h]	Result
1	CHCl_3_/H_2_O	K_2_CO_3_	25	18	No conversion
1	CHCl_3_	DIPEA	25	48	No conversion
1	DMF	Et_3_N	25	144	Observed by NMR‐Difficult purification
1	DMF	Et_3_N	80	2	
1.5	DMF	Et_3_N	80	54	Isolated (8%)

Owing to the different solubilities of the reagents (free base **AMD070** soluble in chloroform, ***p*** **‐SCN‐Bn‐te1pa** as TFA salt soluble in water), a biphasic chloroform/water mixture was first used, not leading to any conversion to the desired product, as evidenced by thin‐layer chromatography (TLC) and ^1^H NMR spectroscopy. Chloroform alone with diisopropylethylamine (DIPEA) as a base did not lead to any conversion either, and the desired **AMD070‐te1pa** was first observed with the DMF/Et_3_N system that was used for further optimization. Although the compound could be identified after reaction at room temperature and 80 °C, purification proved very tedious due to the presence of unreacted ***p*** **‐SCN‐Bn‐te1pa** presenting similar retention times on reversed phase column chromatography. Therefore, the final conditions were set to 80 °C for over two days, with the use of excess **AMD070** to favor full conversion of ***p*** **‐SCN‐Bn‐te1pa** and facilitate the purification step. **AMD070‐te1pa** was thus isolated with high purity (>95% assessed by high performance liquid chromatography ‐ mass spectrometry (HPLC‐MS) detection in very modest 8% yield, but providing enough material (2 mg scale) for further radiolabeling studies. The same conditions were applied to the synthesis and purification of the **AMD070‐benzamide‐te1pa** analog, that required an additional acidic hydrolysis step to cleave the protecting Boc group (Scheme 2), with a total yield of 17% over these two steps (4 mg scale, >99% purity assessed by HPLC‐MS). Both new compounds were fully characterized by means of ^1^H and ^13^C spectroscopies and HRMS.

### 2.2 ^64^Cu Radiolabeling and Cold Copper(II) Complexation

**Figure 1:**
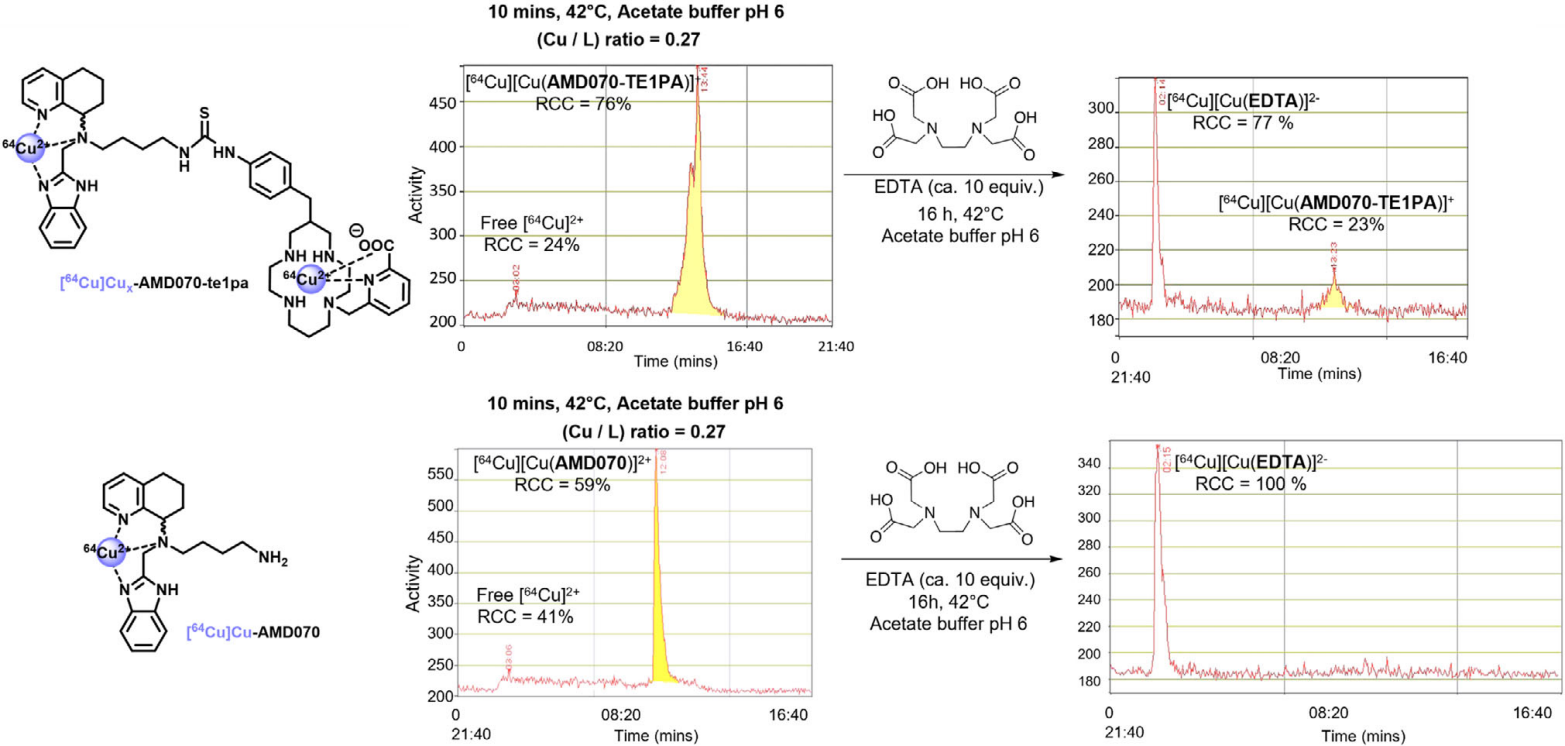
^64^Cu‐radiolabeling of **AMD070‐te1pa** (top) and **AMD070** (bottom) (L: 1800 pmol; Cu: 487 pmol; 7.8 MBq; 10 min, 42 °C, pH 6) and EDTA treatment of nonspecific complexation (20 nmol, 16 h, 42 °C, pH 6).

**Figure 2:**
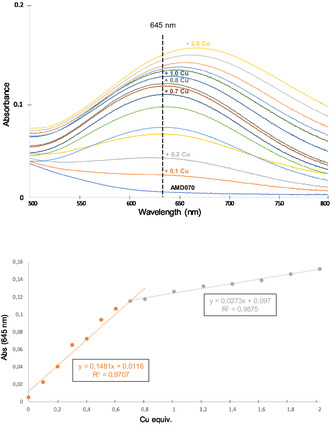
UV–visible titration of **AMD070** by Cu^2+^ ions (top) and absorbance at 645 nm plotted versus Cu^2+^ equiv. (bottom). UV–visible spectrum centered at 650 nm (500–800 nm window) for clarity. Conditions: *C*(**AMD070**) = 1.33 mM; optical path: 1 cm; cuvette volume: 700 μL; total solution volume: 1.200 mL; pH = 3.8.

Radiolabeling conditions were investigated first on the **AMD070‐te1pa** conjugate, starting from previously described method for te1pa–antibody conjugates.^[^ ^40^ ^]^ [^64^Cu] content of the solutions obtained from ARRONAX cyclotron was controlled, with a [Cu]_tot_/[M]_tot_ ratio from (1:4) to (1:8) in the different experiments, the other metals (M) in solution being mainly Ni, Fe, Cu, Co, and Zn as side products from production and decay of [^64^Cu] (see Table S1, Supporting Information). Then **AMD070‐te1pa** (1800 pmol) was placed in sodium acetate buffer (pH 6), [^64^Cu]CuCl_2_ in 0.1 m HCl solution (total Cu : 487 pmol; Activity: 7.8 MBq; total metals: 1800 pmol) was added and the mixture was stirred at 42 °C for 10 min (total solution volume: 155 μL). Radio‐HPLC analysis revealed a radiochemical conversion (RCC) of 76% corresponding to labeling of **AMD070‐te1pa**, alongside 24% of unreacted free [^64^Cu]Cu^2+^ (**Figure** **1**). To complex and remove the latter, excess ethylenediaminetetraacetic acid (EDTA, 20 nmol) was added to the mixture that was kept for 16 h at 42 °C. After this treatment, only 23% of the activity remained associated to the **[** ^**64**^ **Cu]Cu‐AMD070‐te1pa** compound, with 77% corresponding to the **[** ^**64**^ **Cu]Cu‐EDTA** complex. This result was unexpected, as the **te1pa** chelator is well known for its robust complexation of Cu^2+^ ions, in particular with a much higher association constant than EDTA,^[^ ^28^, ^45^ ^]^ and as such transchelation of Cu^2+^ from **te1pa** to EDTA has never been observed previously. Therefore we anticipated that nonspecific coordination of [^64^Cu]Cu^2+^ could occur on the AMD070 fragment, as a kinetically favored species, owing to its potentially coordinating nitrogen atoms. This hypothesis was first verified by submitting **AMD070** to the same labeling conditions (Figure 1, bottom; ligand: 1800 pmol; total Cu: 487 pmol; Activity: 7.8 MBq; total metals: 1800 pmol). In this case, a RCC of 59% was achieved after 10 min at 42 °C, with no remaining associated activity after the treatment with excess EDTA. This suggests that **AMD070** can coordinate copper(II) as a kinetic product in radiolabeling conditions, and that this complexation can be circumvented by transchelation with excess EDTA. This binding could also be demonstrated by UV–visible spectroscopy titrations of **AMD070** by nonradioactive copper(II) solution. Upon addition of increasing amounts of Cu^2+^ ions to a solution of **AMD070** (1.33 mM) in acetate buffer at 25 °C and pH 3.8, a *d–d* transition band characteristic of Cu^2+^ coordination appeared, centered at 645 nm (**Figure** **2**). This signal increased until reaching a plateau around 0.7 equivalents of Cu^2+^. Then a batochromic shift of the *d–d* band was observed with increasing equivalents of copper(II), suggesting the formation of a different species. Absorbance at 645 nm was plotted against Cu^2+^ equivalents (Figure 2), revealing a clear slope change at 0.7 equivalents, consistent with the formation of a L_3_Cu_2_ complex (0.66 Cu/L ratio, L = **AMD070**) that evolves toward lower multiplicity, potentially the LCu complex, in the presence of excess Cu^2+^.

**Figure 3:**
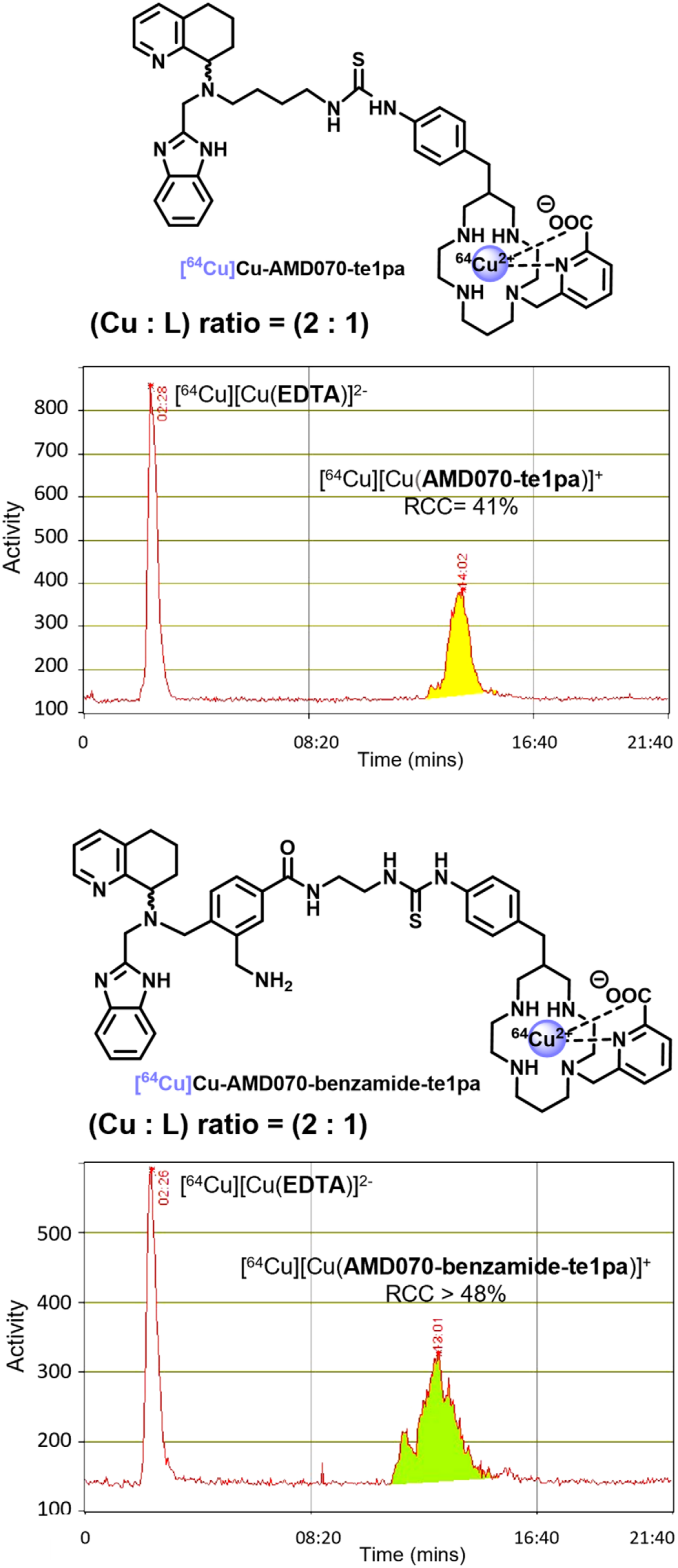
Radio‐HPLC chromatograms of **AMD070‐te1pa** (top) and **AMD070‐benzamide‐te1pa** (bottom) radiolabeling at 95 °C (10 min) with (2:1) Cu/L ratio (ligand: 225 pmol; total Cu: 450 pmol, 29.9 MBq) in sodium acetate buffer (pH 6) followed by treatment with excess EDTA at 95 °C (10 min).

To lower nonspecific binding to the targeting moiety and unforce selective encapsulation of [^64^Cu]Cu^2+^ within the te1pa chelator, that is expected to be the thermodynamically favored species owing to its high affinity constant for copper(II) (log *K* = 25.5),^[^ ^36^ ^]^ radiolabeling temperature was increased to 95 °C for 10 min. This possibility is offered by small organic compounds, in marked contrast with some biomolecules (antibodies in particular) that can be unstable in such harsh radiolabeling conditions. A twofold excess of copper (vs. ligand) was also used to compensate for the residual nonspecific binding (ligand: 225 pmol; total Cu: 450 pmol; Activity: 29.9 MBq; total metals: 3400 pmol). This labeling step was followed by a treatment with excess EDTA (20 nmol) for 10 min at 95 °C, which resulted in high RCC for both compounds (**Figure** **3**): 41% for **AMD070‐te1pa** and over 48% for **AMD070‐benzamide‐te1pa** (theoretical maximal RCC = 50% owing to the ligand‐to‐metal ratio = 0.5). Through this step, only residual copper(II) linked to the AMD070 motif is trapped by EDTA as the Cu(II)–te1pa complex is sufficiently inert. Finally, the separation of the prepared radiopharmaceuticals from the [^64^Cu]Cu–EDTA complex could be easily achieved on C_18_‐silica Sep‐Pak cartridges. Indeed, the EDTA complex was first eluted with aqueous NaCl, then the desired products could be obtained by elution with a 1:1 aqueous NaCl/ethanol mixture. Subsequent evaporation of the solvents can then lead to injectable solutions suitable for *in vitro* and *in vivo* studies.

## 3 Conclusion

Finally, we have successfully prepared two conjugates of the small molecule **AMD070**, including an efficient copper chelator in the structure. Two different architectures have been selected, one via direct derivatization of **AMD070** terminal amine function leading to the **AMD070‐te1pa** analog, and the other through the use of the corresponding benzamide derivative leading to **AMD070‐benzamide‐te1pa**. The ^64^Cu‐radiolabeling of these two molecules has been fully optimized to avoid nonspecific fixation of copper on the AMD070 motif, and excellent radiochemical conversions could be obtained (41% and >48%, respectively, with respect to the 2 equiv. of Cu and conversion of 82% and >96%, respectively, with respect to ligand). This will now allow the study of the CXCR4‐recognition properties (*in vitro* and *in vivo*) of these two novel radiopharmaceuticals that are, to the best of our knowledge, the first ^64^Cu‐radiolabeled AMD070 derivatives and hold a strong potential for CXCR4‐targeted PET imaging.

## Conflict of Interest

The authors declare no conflict of interest.

## Author Contributions

**Marie M. Le Roy**: investigation (lead); writing—review & editing (supporting). **Patricia Le Saëc**: investigation (equal); writing—review & editing (supporting). **Michel Chérel**: data curation (equal); supervision (equal); writing—review & editing (supporting). **Alain Faivre‐Chauvet**: conceptualization (equal); supervision (equal). **Raphaël Tripier**: conceptualization (equal); funding acquisition (equal); supervision (equal); writing—review & editing (equal). **Thibault Troadec**: data curation (equal); funding acquisition (equal); project administration (lead); resources (lead); supervision (equal); writing—original draft (lead); writing—review & editing (lead).

## Supporting information

Supplementary Material

## Data Availability

The data that support the findings of this study are available in the supplementary material of this article.
