# Supplementary material for: Synthesis and 64Cu‐Radiolabeling Strategies of Small Organic Radioconjugates Based on the AMD070 Scaffold
Source: ChemMedChem. 2025 Jun 14;20(15):e202500243. doi: 10.1002/cmdc.202500243 (PMC12321274; doi:10.1002/cmdc.202500243)
Supplement: Supplementary file 1 — Supplementary Material [file CMDC-20-e202500243-s001.pdf]

Synthesis and ^{64}Cu-Radiolabeling Strategies of Small Organic Radioconjugates Based on the AMD070 Scaffold.

Marie M. Le Roy,[a] Patricia Le Saëc,[b] Michel Chérel,[b,c] Alain Faivre-Chauvet,[b,d] Thibault Troadec*[a] and Raphaël Tripier*[a]

[a] *Univ. Brest, UMR CNRS 6521 CEMCA, 6 avenue Le Gorgeu, 29200 Brest, France.*
[b] *Univ Angers, Nantes Univ, Inserm, CNRS,CRCI2NA,CHU Nantes, F-44007 Nantes, France.*
[c] *Institut de Cancérologie de l'Ouest, F-44800 Saint-Herblain, France.*
[d] *Nuclear Medicine Department, University Hospital, Nantes, France.*

Electronic Supporting Information

General Considerations

Reagents and solvents

Reagents used for synthesis were purchased from SIGMA-ALDRICH®, TCI chemicals®, ACROS ORGANICS® and Ambeed® and used without further purification. Solvents for synthesis were obtained from a MBraun MB-SPS 800 purification system. Ultrapure water was freshly obtained from a Milli-Q dispenser. **AMD070** and ***p*-SCN-Bn-te1pa** were prepared according to literature.[1,2] Compound **1** was prepared via an procedure adapted from literature.[3]

Nuclear Magnetic Resonance Spectroscopy

NMR data were recorded at the "service commun" of the Université de Bretagne Occidentale. ^{1}H, ^{13}C and 2D NMR spectra were recorded on a Bruker Avance III HD 500 (500.25 MHz for 1H and 125.79 MHz for ^{13}C) or Bruker AMX-3 300 (300.13 MHz for ^{1}H and 75.47 MHz for ^{13}C) spectrometers. Deuterated solvents from Eurisotop® are used to reference spectra, ^{1}H and ^{13}C shifts are reported in ppm and the δ scales are relative to TMS.

The signals are indicated as follows: chemical shift, multiplicity (s for singlet; br for broad singlet, d for doublet; t for triplet; q for quadruplet; m for multiplet), coupling constants J in Hertz (Hz), assignment: $CH_2\alpha N$, $CH_2\beta N$ or $CH_2\gamma N$ correspond to CH_2 located in alpha, beta or gamma position of considered nitrogen atom, type of nuclei is indicated in italic. Ar is a generic term used in subscript for all H or C aromatic atoms.

Mass Spectrometry

High-Resolution Mass Spectrometry (HRMS) analyses were performed at the Institute of Organic and Analytic Chemistry (ICOA Orléans) on a HRMS Q-Tof MaXis.

High Performance Liquid Chromatography

The analytic HPLC analyses were performed on a Prominence Shimadzu HPLC/LCMS-2020 instrument equipped with an UV SPD-20 A detector. The chromatographic system employs C18AQ 5 µM 250 × 4.6 mm columns with H2O (0.1% TFA) – MeCN or MeOH (v/v) as eluents at a flow rate of 1 mL/min and UV detection at 254 and 350 nm.

UV-Vis and Kinetic Inertness Studies

UV-Vis spectra were recorded at 25°C on a JASCO V-760 spectrometer equipped with a PAC-743R Peltier temperature control device in 700 μL cuvettes with 1 cm optical path, and a baseline correction was applied.

For Cu^{2+} complexation with **AMD070**, 15 solutions containing from 0 to 2.0 eq. Cu^{2+} were prepared from 800 μL of **AMD070** stock solution (at C = 2 mM) in acetate buffer (pH 3.8), Cu^{2+} aliquot from stock solution (standardized at C = 52.2 mM in pure water) and completed to 1.200 mL with acetate buffer (pH 3.8). Solutions were equilibrated for 1h at 25°C prior to UV-Vis measurements.

Synthesis of new compounds

Compound 1

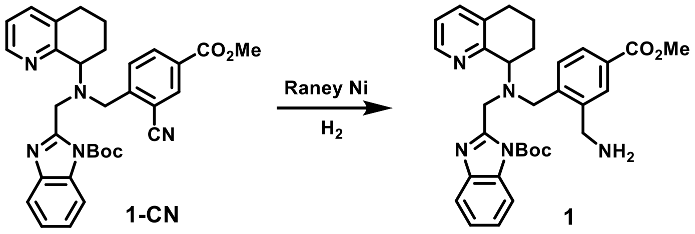

Formula: $C_{32}H_{37}N_5O_4$
Molecular weight: 555.28 g.mol^{-1}
Description: yellow powder
Yield: 32%

1-CN (0.286 g, 0.52 mmol, from ref[3]) in NH_3MeOH (7N, 25 mL), was treated with Raney Ni (~10 g) and placed under 5 bars of H_2 in an autoclave for 18h at 25°C. The mixture was diluted with MeOH, filtered through Celite® 521, washed with MeOH and the combined filtrate was concentrated. The crude was purified by column chromatography on activated neutral alumina (100:0 to 90:10 CH_2Cl_2/MeOH) to afford **1** as a yellow powder (0.090 g, 32%).

^{1}H NMR (MeOD, 400 MHz, 298 K) δ (ppm) 8.58 (s, 1H, CH_{Ar}), 7.82 (s, 1H, CH_{Ar}), 7.64 (d, 1H, CH_{Ar}, *J* = 7.6 Hz), 7.48 (d, 2H, CH_{Ar}, *J* = 7.6 Hz), 7.42 (m, 2H, CH_{Ar}), 7.17 (q, 1H, CH_{Ar} *J* = 4.8, 7.7 Hz), 7.12 (m, 2H, CH_{Ar}), 4.38 (d, 1H, CH_2-NH_2, *J* = 15.6 Hz), 4.28 (d, 1H, CH_2-NH_2, *J* = 15.6 Hz), 4.08 (m, 1H, C*H*), 4.01-3.85 (m, 4H, CH_2), 3.80 (s, 3H, O-CH_3), 2.90-2.79 (m, 1H, CH_2), 2.75-2.67 (m, 1H, CH_2), 2.29 (m, 1H, CH_2), 2.06 (m, 2H, CH_2), 1.67 (m, 1H, CH_2), 1.48 (s, 9H, CH_3Boc)

^{13}C Jmod NMR (MeOD, 100 MHz, 298 K) δ (ppm)168.1 (*C*O-CH_3), [158.4, 157.7] (C_{ipso}), 155.2 (CO_{Boc}), 148.1 (CH_{Ar}), [143.0, 140.3] (C_{ipso}), 138.7 (CH_{Ar}), 136.4 (C_{ipso}), 130.2 (C_{ipso}), [131.4, 130.0, 128.7, 123.4, 123.2] (CH_{Ar}), 80.2 (C_{quat}Boc), 62.9 (*C*H), 54.7 (N-CH_2-Ar), 52.4 (O-CH_3), 50.4 (N-CH_2-benzimidazole), 42.1 ($CH_2\alpha NH_2$), 28.9 (CH_3Boc), [30.0, 24.8] (CH_2), 22.5 ($CH_2\gamma$N)

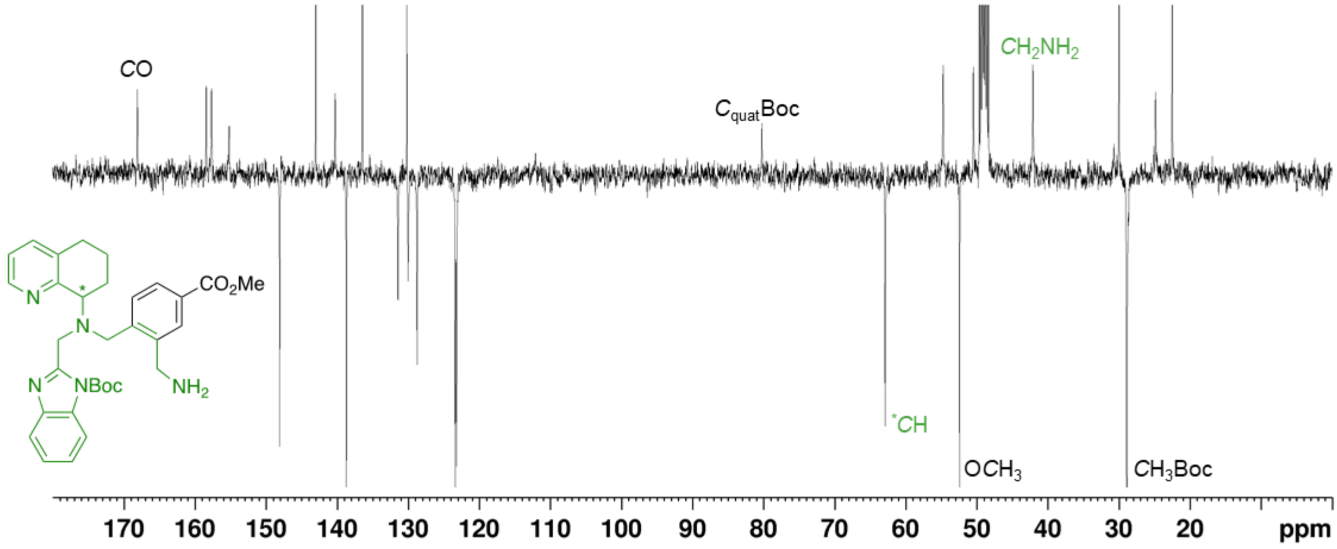

Compound 2

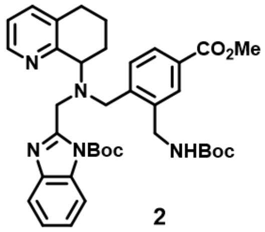

2

Formula: $C_{37}H_{45}N_5O_6$
Molecular weight: 655.34 g.mol^{-1}
Description: white foam
Yield: 94%

Compound **1** (0.090 g, 0.16 mmol) was dissolved in THF (8 mL). Di-*tert*-butyl dicarbonate (0.053 g, 0.24 mmol, 1.5 eq) was added to the solution and the reaction was stirred under reflux for 18h. The solvent was removed under reduced pressure. The crude product was purified by column chromatography on silica gel (100:0 to 98:2 CH_2Cl_2/MeOH) to afford **2** as a white foam (0.099 g, 94%).

^{1}H NMR (MeOD, 300 MHz, 298 K) δ (ppm) 8.21 (s, 1H, CH_{Ar}), 7.90 (s, 1H, CH_{Ar}), 7.73-7.65 (m, 2H, CH_{Ar}), 7.45-7.37 (m, 2H, CH_{Ar}), 7.26-7.15 (m, 2H, CH_{Ar}), 7.12 (d, 1H, CH_{Ar}), 6.75 (m, 1H, CH_{Ar}), 4.55-4.24 (m, 6H, CH_2), 3.92 (m, 1H, C*H*), 3.81 (s, 3H, O-CH_3), 2.84-2.67 (m, 1H, CH_2), 2.61-2.48 (m, 1H, CH_2), 2.25-2.08 (m, 2H, CH_2), 2.00 (m, 1H, CH_2), 1.70 (s, 9H, CH_3Boc), 1.39 (s, 9H, CH_3Boc)

^{13}C Jmod NMR (MeOD, 75 MHz, 298 K) δ (ppm) 168.1 (*C*O), [158.5, 158.2] (C_{ipso}), 155.3 (*C*OBoc), 149.6 (*C*OBoc), 147.4 (CH$_{Ar}$), [143.8, 142.1, 141.2] (C_{ipso}), 137.5 (CH$_{Ar}$), [135.5, 133.9] (C_{ipso}), [131.7, 131.4] (CH$_{Ar}$), 130.4 (C_{ipso}), [128.8, 125.7, 125.0, 122.0, 120.1, 115.9] (CH$_{Ar}$), [86.8, 79.8] (C_{quat}Boc), 61.7 (N-*C*H), 56.6 (N-*C*H$_2$-Ar), 52.4 (*C*H$_3$), 52.3 (N-*C*H$_2$-benzimidazole), 42.2 (*C*H$_2$-NHBoc), 30.0 (*C*H$_2$), [28.9, 28.4] (*C*H$_3$Boc), 25.2 (*C*H$_2$), 22.8 (*C*H$_2\gamma$N)

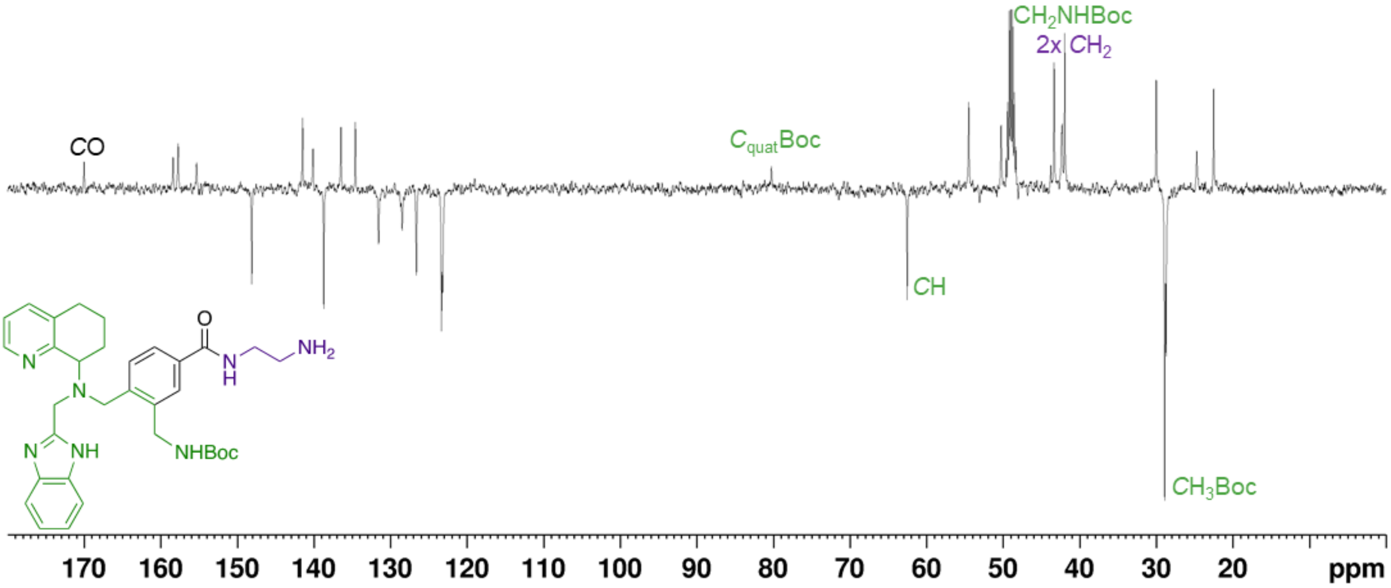

ESI (positive, H_2O) m/z calcd. for $[C_{37}H_{46}N_5O_6]^+$ 656.344; found $[M+H]^+$ 656.350

Compound 3

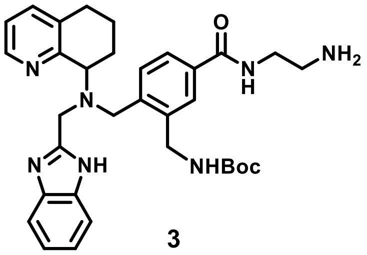

3

Formula: $C_{38}H_{49}N_7O_5$
Molecular weight: 583.74 g.mol^{-1}
Description: brown powder
Yield: 86%

Compound 2 (0.099 g, 0.15 mmol) was dissolved in an excess of ethylenediamine (16 mL), and the reaction was stirred at room temperature for 5 days. The solvent was removed under reduced pressure. The residue was redissolved in $CHCl_3$, and the organic layer was washed with H_2O, dried over $MgSO_4$, filtered, and concentrated *in vacuo* to afford compound **3** as a brown powder (0.076 g, 86%).

^{1}H NMR (MeOD, 300 MHz, 298 K) δ (ppm) 8.56 (s, 1H, CH_{Ar}), 7.66 (s, 1H, CH_{Ar}), 7.56-7.39 (m, 5H, CH_{Ar}), 7.17-7.09 (m, 3H, CH_{Ar}), 4.36 (d, 1H, CH_2, J = 14.0 Hz), 4.24 (d, 1H, CH_2, J = 14.0 Hz), 4.04-3.97 (m, 1H, CH), 3.97-3.84 (m, 4H, CH_2), 3.38 (t, 2H, CH_2, J = 6.32 Hz), 3.10 (t,eda), 2.86-2.76 (t, 3H, CH_2, J = 6.32 Hz), 2.71-2.61 (m, 2H, CH_2), 2.23 (m, 1H, CH_2), 2.07-1.95 (m, 2H, CH_2), 1.66-1.56 (m, 1H, CH_2), 1.49-1.39 (m, 9H, CH_3Boc)

^{13}C Jmod NMR (MeOD, 100 MHz, 298 K) δ (ppm) 170.1 (CO), [158.4, 157.7, 155.3] (C_{ipso}), 148.1 (CH$_{Ar}$), [141.5, 140.1] (C_{ipso}), 138.7 (CH$_{Ar}$), [136.5 134.6] (C_{ipso}), [131.6, 128.5, 126.6, 123.4, 123.2] (CH$_{Ar}$), 80.3 (C_{quat}Boc), 62.6 (CH), 54.5 (N-CH$_2$-Ar), 49.8 (N-CH$_2$-benzimidazole), [43.4, 42.3, 42.0] (NH-CH$_2$), 30.0 (CH$_2$), 28.8 (CH$_3$Boc), 24.7 (CH$_2$), 22.5 (CH$_2\gamma$N)

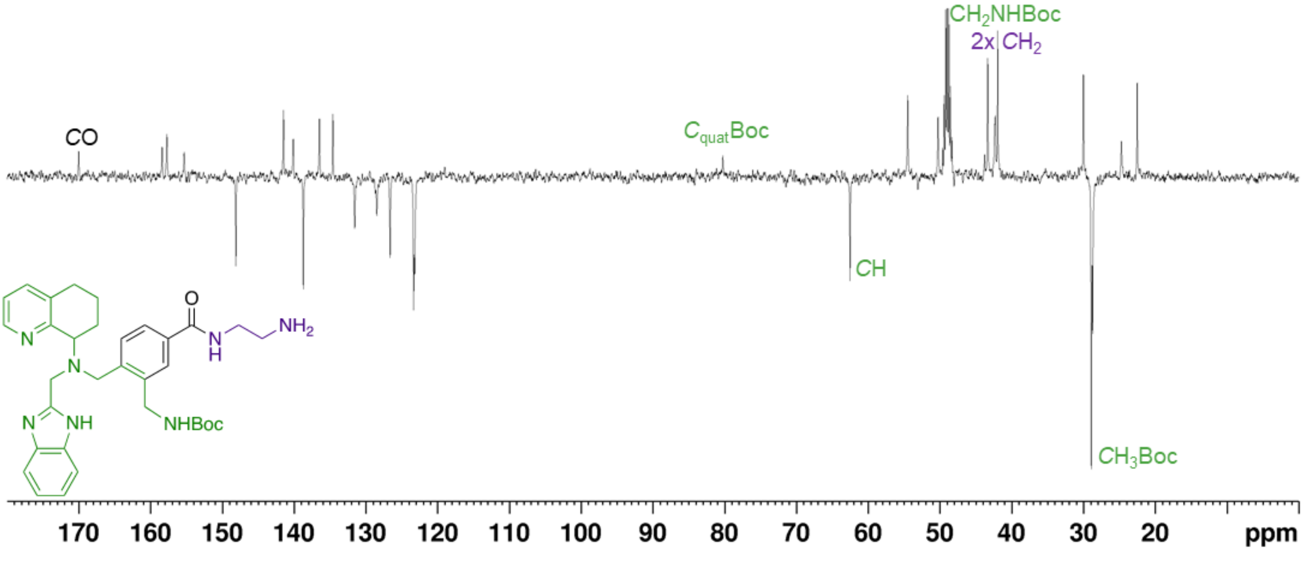

HRMS (ESI, positive, H_2O) m/z calcd. for $[C_{33}H_{42}N_7O_3]^+$ 584.3344 found $[M+H]^+$ 584.3338, calcd. for $[C_{33}H_{41}N_7NaO_3]^+$ 606.3163 found $[M+Na]^+$ 606.3158, calcd. for $[C_{33}H_{43}N_7O_3]^{2+}$ 292.6708 found $[M+2H]^{2+}$ 292.6709

Melting point: 84°C

AMD070-TE1PA

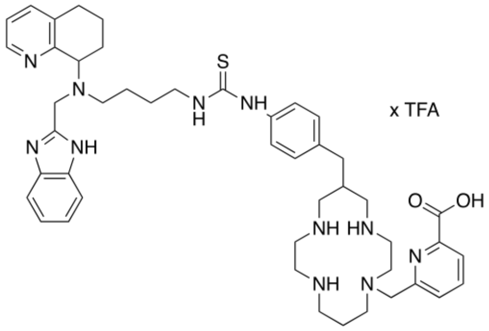

Formula: $C_{46}H_{61}N_{11}O_2S$
Molecular weight: 832.13 g.mol^{-1} (x = 0)
1288.22 g.mol^{-1} (x = 4)
Description: off-white foam
Yield: 8 % (for x = 4)

To a solution of **AMD070** (10.5 mg, 0.03 mmol) and ***p*-SCN-Bn-TE1PA**.3TFA (20.0 mg, 0.02 mmol) in distilled DMF (0.5 mL) triethylamine (0.011 mL, 0.08 mmol) was added. The reaction mixture was heated at 80°C for 2.5 days. The solvent was removed under reduced pressure. The crude product was purified by flash chromatography on C18 reversed-phase (H_2O + 0.1% TFA/MeOH; 100:0 to 0:100) to afford **AMD070-TE1PA**.4TFA as an off-white foam after lyophilization (1.98 mg, 8%).

^{1}H NMR (MeOD, 500 MHz, 298K) δ (ppm) 8.83 (s, 1H, CH_{Ar}), 8.31 (s, 1H, CH_{Ar}), 8.16 (br, 1H, CH_{Ar}), 7.99 (br, 1H, CH_{Ar}), 7.86-7.80 (m, 4H, CH_{Ar}), 7.61-7.54 (m, 4H, CH_{Ar}), 7.25 (br, 2H, CH_{Ar}), 4.54-4.39 (m, 4H), 4.27 (br, 1H), 3.48 (br, 5H), 3.20-2.16 (br, 33H), 2.04 (br, 2H), 1.57 (br, 3H)

^{13}C Jmod NMR (MeOD, 125 MHz, 298K) δ (ppm) 182.3 (*C*S), 168.4 (*C*O), [159.5, 153.5, 153.1, 149.9] (C_{ipso}), [147.9, 141.5] (*C*H_{Ar}), 141.0 (C_{ipso}), 140.0 (*C*H_{Ar}), [136.7, 132.9] (C_{ipso}), [130.6, 129.9, 128.9, 127.4, 126.6, 126.4, 125.7, 125.1, 115.2] (*C*H_{Ar}), 61.2 (*C*H), [58.1, 56.9, 54.7, 54.4, 53.3, 52.7, 47.2, 46.8, 46.1, 44.8, 44.6] (*C*$H_2\alpha$N), 38.6 (*C*H), [28.8, 27.7, 26.1, 23.6, 21.5, 21.4] (*C*H_2)

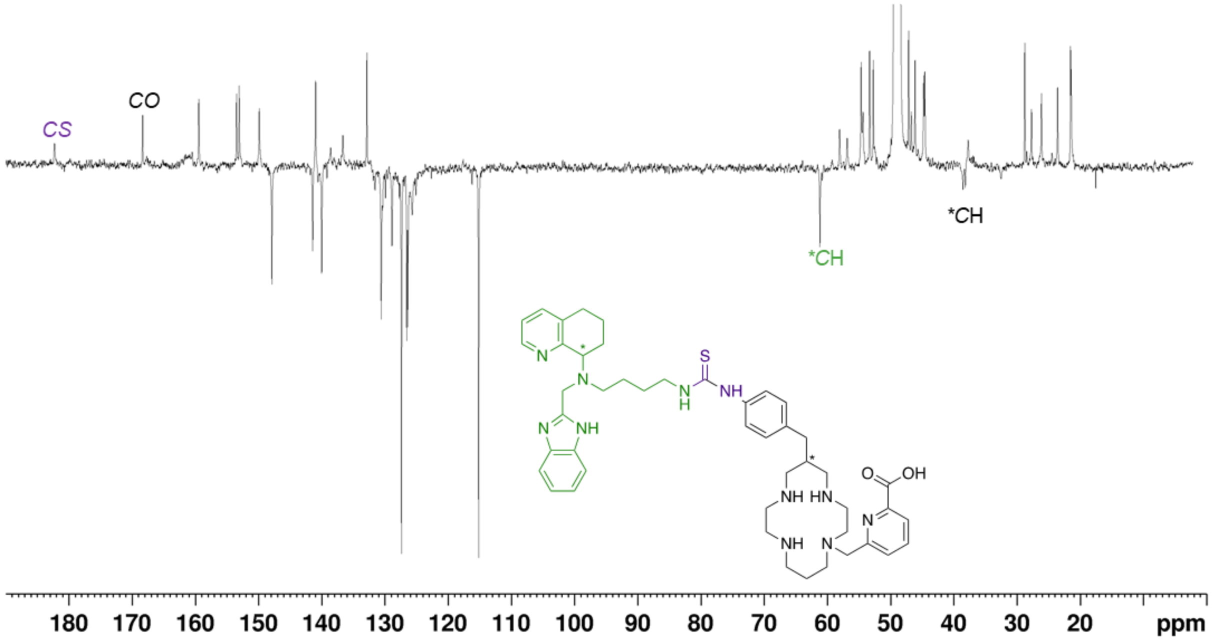

HRMS (ESI, positive, H_2O) m/z calcd. for $[C_{46}H_{62}N_{11}O_2S]^+$ 832.4803 found $[M+H]^+$ 832.4817, calcd. for $[C_{46}H_{61}N_{11}NaO_2S]^+$ 854.4623 found $[M+Na]^+$ 854.4625, calcd. for $[C_{46}H_{63}N_{11}O_2S]^{2+}$ 416.7438 found $[M+2H]^{2+}$ 416.7458

Analytical HPLC, reversed-phase, (C-18 silica, H_2O 0.1% Formic acid/ MeOH 95:5 to 10:90): t_R=19.43 min, 95% purity

AMD070-benzamide-TE1PA

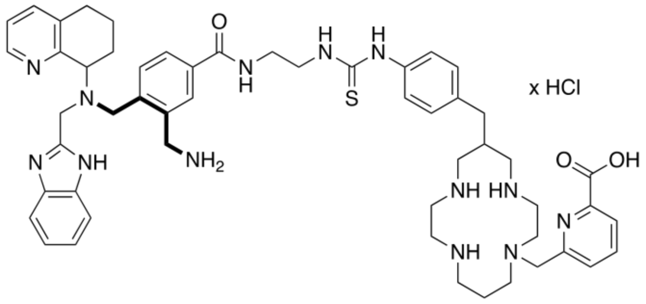

Formula: $C_{53}H_{67}N_{13}O_3S$
Molecular weight: 966.27 g.mol^{-1} (x = 0)
1148.56 g.mol^{-1} (x = 5)
Description: off-white foam
Yield: 17% over 2 steps

To a solution of **3** (20 mg, 0.03 mmol) and ***p*-SCN-Bn-TE1PA**.3TFA (20 mg, 0.02 mmol) in distilled DMF (0.5 mL), triethylamine (0.011 mL, 0.08 mmol) was added. The reaction mixture was heated at 80°C for 2.5 days. The solvent was removed under reduced pressure. The crude product was dissolved in 1 mL of HCl (1 M) and the reaction was stirred at room temperature overnight. The solvent was removed under reduced pressure. The crude product was purified by precipitation (water/acetone) to afford compound **AMD070-benzamide-TE1PA**.5HCl as an off-white foam after lyophilization (4.0 mg, 17% over 2 steps).

^{1}H NMR (D_2O, 500 MHz, 298 K) δ (ppm) 8.82 (s, 1H, CH_{Ar}), 8.45 (s, 1H, CH_{Ar}), 8.25-7.92 (m, 4H, CH_{Ar}), 7.64-7.11 (m, 11H, CH_{Ar}), 4.61-4.46 (m, 2H, CH_2), 4.40-4.23 (m, 3H, CH_2), 4.09 (br, 1H, CH), 4.04-3.51 (br, 6H, CH_2), 3.51-3.29 (br, 4H, CH_2), 3.27-2.89 (br, 11H, CH_2), 2.82-2.42 (m, 6H, CH_2), 2.37-2.21 (br, 3H, CH_2), 2.21-1.86 (m, 4H, CH_2)

^{13}C Jmod NMR (D_2O, 125 MHz, 298 K) δ (ppm) 182.6 (*C*S), [171.1, 170.8] (*C*O), [160.4, 153.1, 152.4] (C_{ipso}), 151.0 (CH$_{Ar}$), 143.8 (C_{ipso}), [142.5, 142.2] (CH$_{Ar}$), [142.1, 142.0, 140.6, 135.9, 135.3] (C_{ipso}), [135.0, 134.9] (CH$_{Ar}$), [134.7, 134.6] (C_{ipso}), [133.5, 133.2] (CH$_{Ar}$), 132.7 (C_{ipso}), [131.6, 131.4, 131.3, 130.9, 130.6, 130.2, 129.6, 129.4, 129.3, 128.7, 127.9, 126.0, 123.0, 116.3, 116.3] (CH$_{Ar}$), 65.5 (*C*H), [59.6, 57.1, 56.3, 55.7, 54.7, 51.4, 48.7, 48.5, 47.7, 47.2, 46.1, 42.5, 42.1, 41.8, 40.0] (CH$_2\alpha$N), 39.3 (*C*H), [38.7, 30.3] (CH$_2$), 25.0 (CH$_2\beta$N), 23.5 (CH$_2$)

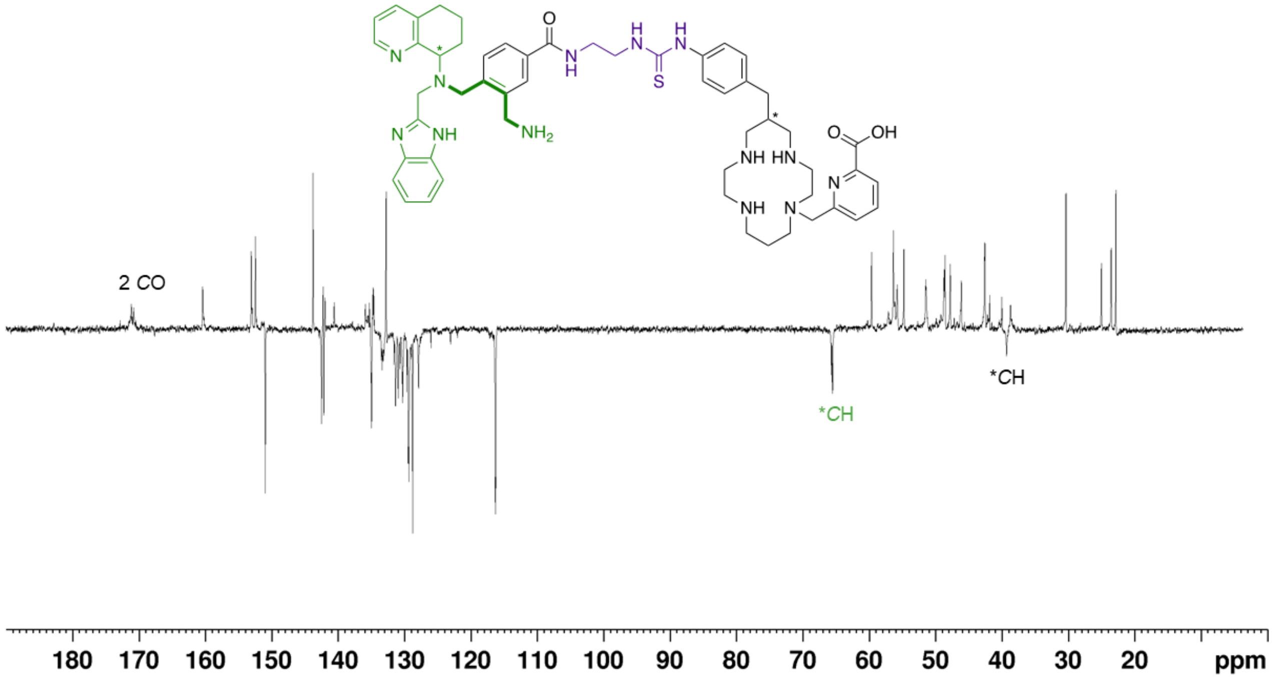

HRMS (ESI, positive, H_2O) m/z calcd. for $[C_{53}H_{68}N_{13}O_3S]^+$ 966.5283 found $[M+H]^+$ 966.5264, calcd. for $[C_{53}H_{69}N_{13}O_3S]^{2+}$ 483.7678 found $[M+2H]^{2+}$ 483.7700

Analytical HPLC, reversed-phase, (C-18 silica, H_2O 0.1% Formic acid/ MeOH 95:5 to 10:90): t_R=8.31, > 99 % purity

Radiolabeling studies

[^{64}Cu] was obtained from the ARRONAX cyclotron (Saint-Herblain, France) with production by the (^{64}Ni(d,2n)) route from enriched [^{64}Ni] (min. 98%) electrodeposited on gold solid support, and provided as [^{64}Cu]$CuCl_2$ in 0.1 M HCl. Radionuclidic purity was determined by gamma spectroscopy using a DSPEC-JR-2.0 type 98-24B HPGE detector (AMETEK) and chemical purity was controlled by ICP-OES with an iCAP 6500 DUO (Thermo Fisher Scientific).

Table S1 : Example of radionuclide purity and chemical purity of copper-64 products used in experiments

Radionuclides	Radioactive concentration at calibration date (MBq/mL) *Target 1*	Radioactive concentration at calibration date (MBq/mL) *Target 2*	Metals	Concentration of stock solution (ppm) *Target 1*	Concentration of stock solution (ppm) *Target 2*
^{64}Cu	16.30E2 ± 0.78E2	17.79E2 ± 0.76E2	Co	0.325 ± 0.031	0.1608 ± 0.0029
^{65}Ni	< 5.88E-3	< 10.99E-3	Cu	1.03 ± 0.18	1.242 ± 0.012
^{57}Co	< 11.66E-1	< 21.28E-1	Fe	<0.46	0.539 ± 0.041
^{58}Co	< 25.34E-2	< 46.96E-2	Ni	0.437 ± 0.012	3.736 ± 0.097
^{61}Co	< 15.82E-4	< 12.10E-4	Zn	1.491 ± 0.016	3.4320 ± 0.0085

Radiolabeling optimized conditions : to neutralize hydrochloride ions and maintain a pH close to 6, acetate buffer was added to [^{64}Cu]$CuCl_2$ solution. **AMD070-te1pa** and **AMD070-benzamide-te1pa** (225 pmol) were radiolabeled by addition of [^{64}Cu]$CuCl_2$ (450 pmol, 29.9 MBq) and incubation at 95°C during 10 min (total solution volume : 155µL). Then, a calculated volume of 1 mM EDTA was added to the radiolabeling solution to complex free ^{64}Cu. The mixture was stirred at 95°C during 10 min. Purification on SepPak C18 (NaCl/EtOH) allows the separation of the radiocomplex [^{64}Cu][Cu(**EDTA**)]$^{2-}$ from the radiocomplexes [^{64}Cu][Cu(**AMD070- te1pa**)] and [^{64}Cu][Cu(**AMD070-benzamide- te1pa**)]. The radiochemical purity was evaluated by injection of the radiolabeled solution into a radio-HPLC system (Eckert & Ziegler HPLC module Modular Lab software and detector; stationary phase ACE C18 column; mobile phase (0.05% TFA in $H_2$0 : Acetonitrile), (90:10) to (0:100)) and the integration of the corresponding peak areas allowed calculation of the radiochemical conversions. All labeling experiments were conducted in duplicates.

Radiochemical conversions (RCCs) were calculated by integration of HPLC signals, assuming that no activity remains on the column, and must be interpreted with care.

References

1 United States, US20080171740A1, 2008.
2 Z. Halime, M. Frindel, N. Camus, P.-Y. Orain, M. Lacombe, M. Chérel, J.-F. Gestin, A. Faivre-Chauvet and R. Tripier, New synthesis of phenyl-isothiocyanate C-functionalised cyclams. Bioconjugation and 64Cu phenotypic PET imaging studies of multiple myeloma with the te2a derivative, Org. Biomol. Chem., 2015, 13, 11302–11314.
3 R. Skerlj, G. Bridger, E. McEachern, C. Harwig, C. Smith, T. Wilson, D. Veale, H. Yee, J. Crawford, K. Skupinska, R. Wauthy, W. Yang, Y. Zhu, D. Bogucki, M. Di Fluri, J. Langille, D. Huskens, E. De Clercq and D. Schols, Synthesis and SAR of novel CXCR4 antagonists that are

potent inhibitors of T tropic (X4) HIV-1 replication, Bioorg. Med. Chem. Lett., 2011, 21, 262–266.
